# 2727. Risk factors and clinical outcomes of tuberculosis among hematopoietic stem cell transplant recipients in endemic country: A single-center, case-control study

**DOI:** 10.1093/ofid/ofad500.2338

**Published:** 2023-11-27

**Authors:** Kamolchanok Tanchotikul, Tuntanut Lohawatcharagul, Kamonwan Jutivorakool, Udomsak Bunworasate, Jakapat Vanichanan

**Affiliations:** Faculty of Medicine, Chulalongkorn University and King Chulalongkorn Memorial Hospital, Bangkok, Krung Thep, Thailand; Faculty of Medicine, Chulalongkorn University and King Chulalongkorn Memorial Hospital, Bangkok, Krung Thep, Thailand; King Chulalonkorn Memorial Hospital, Bangkok, Krung Thep, Thailand; Faculty of Medicine, Chulalongkorn University and King Chulalongkorn Memorial Hospital, Bangkok, Krung Thep, Thailand; Chulalongkorn University, Bangkok, Krung Thep, Thailand

## Abstract

**Background:**

Tuberculosis (TB) is an infectious disease caused by *Mycobacterium tuberculosis* following reactivation from latent infection. Immunocompromised individuals, including hematopoietic stem cell transplant (HSCT) recipient, are at risk for active TB which can lead to poor outcomes. Currently, the epidemiological data and risk factors related to TB infection among HSCT recipients especially in endemic country are still limited.

**Methods:**

A matched single-center, case-control study was conducted in King Chulalongkorn Memorial Hospital. Cases were defined as HSCT recipients who were diagnosed proven or probable active TB between January 2008 to December 2020. For each case, 5 controls were matched by gender, age (±5 years), and type of transplant. Risk factor associated with TB was determined using multivariate cox regression.

**Results:**

During the study period, a total of 570 patients underwent HSCT. Eleven patients were diagnosed with active TB represented incidence of 1.93%. Pulmonary involvement was the most common presentation (64%) followed by pleura (18 %), pleuropulmonary (9%) and pericardium (9%). Active TB was diagnosed at median duration of 3.76 months (1.33-22.88) after HSCT. Overall median duration for treatment was 6 months (IQR, 1.2-11.5). Autologous HSCT recipients mostly received rifampin-based regimen while allogeneic HSCT recipients received quinolone-containing regimen. Eight patients (73%) were cured, while another 3 (27%) patients died. There was no significant difference in mortality and primary disease relapse between case and control group. In multivariate analysis, previous TB contact or infection (OR 5.852 95%CI 1.268-27.011) and invasive aspergillosis (OR 6.113 95%CI 1.467-25.475) were associated with active TB in HSCT recipient.

**Conclusion:**

Incidence and mortality of TB among HSCT recipients were higher compared to general population. Active TB was diagnosed shortly after HSCT and previous TB exposure was a significant risk factor. These findings may suggest reactivation of latent TB is the main pathogenesis of this patient population in endemic country.

**Disclosures:**

**All Authors**: No reported disclosures

